# True Fibroma of Alveolar Mucosa

**DOI:** 10.1155/2014/904098

**Published:** 2014-03-04

**Authors:** Shankargouda Patil, Roopa S. Rao, Sanketh Sharath, Anveeta Agarwal

**Affiliations:** Department of Oral Pathology, M.S. Ramaiah Dental College, Bangalore 54, India

## Abstract

Benign fibrous overgrowths are often found in the oral cavity, almost always being reactive/irritational in nature. However, benign mesenchymal neoplasms of the fibroblasts are extremely uncommon. Here we report a case of “True Fibroma of Alveolar Mucosa” for its rarity.

## 1. Introduction

Fibroma is a benign neoplasm of fibroblastic origin, rare in the oral cavity [1]. It is said that the majority of the fibromas occurring in the oral cavity are reactive in nature and represent a reactive hyperplasia of fibrous connective tissue in response to local irritation or trauma rather than being a true neoplasm [2]. The occurrence of irritation fibromas among the South Indian population was found to be 39.1% [3].

Stout said that “it is exceedingly difficult to decide whether or not there is true benign neoplasm composed of fibroblasts” [2]. Since true fibromas of oral and maxillofacial areas are infrequent [4], the case below is one of a kind.

The histologic criteria of a true fibroma were first described by Barker and Lucas. Three more cases of true gingival fibroma have been reported in literature since then [5]. We describe a similar case of a true fibroma in the maxillary alveolar mucosa.

## 2. Case Report

**Figure 1:**
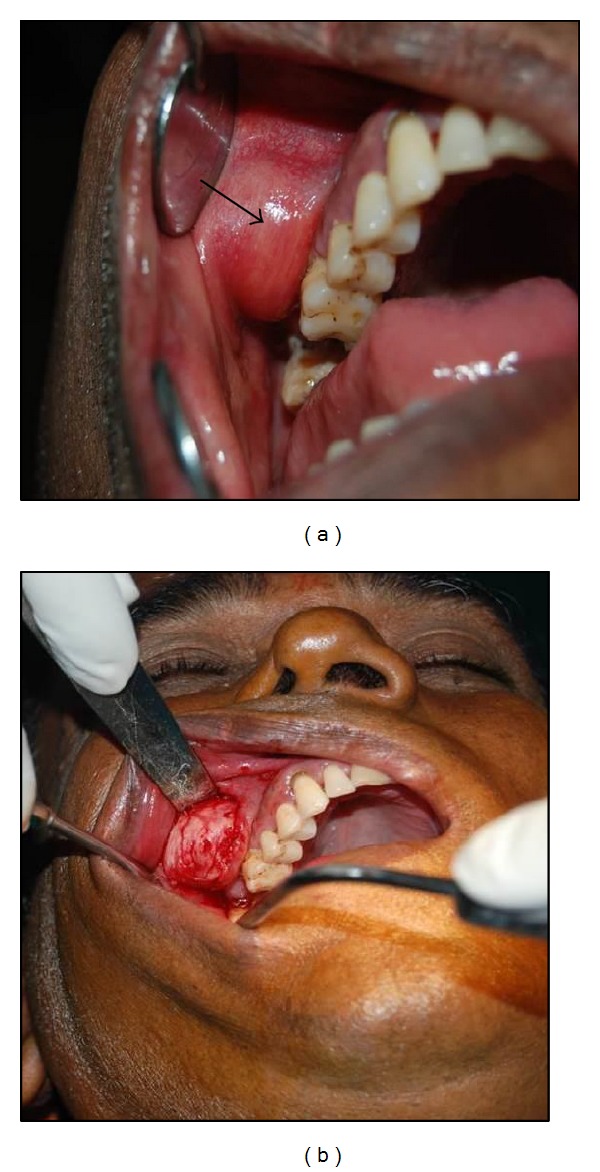
(a) Well circumscribed lesion in the alveolar mucosa. (b) Encapsulated tumour on surgical exploration.

A 60-year-old male was examined for a two-month-old solitary swelling measuring approximately 2.5 × 2.5 cm in the right middle third of the face. Intraorally, on palpation it was ovoid and firm in consistency with respect to 16 and 17 region (Figure 1(a)). Radiographic examination revealed no calcifications. Irritation fibroma, neurofibroma, peripheral ossifying fibroma, and benign tumors of nerve and muscle origin were provisionally diagnosed. The encapsulated lesion, noted to be located between periosteum and alveolar mucosa, was excised under local anaesthesia and sent for histopathological evaluation (Figure 1(b)).

**Figure 2:**
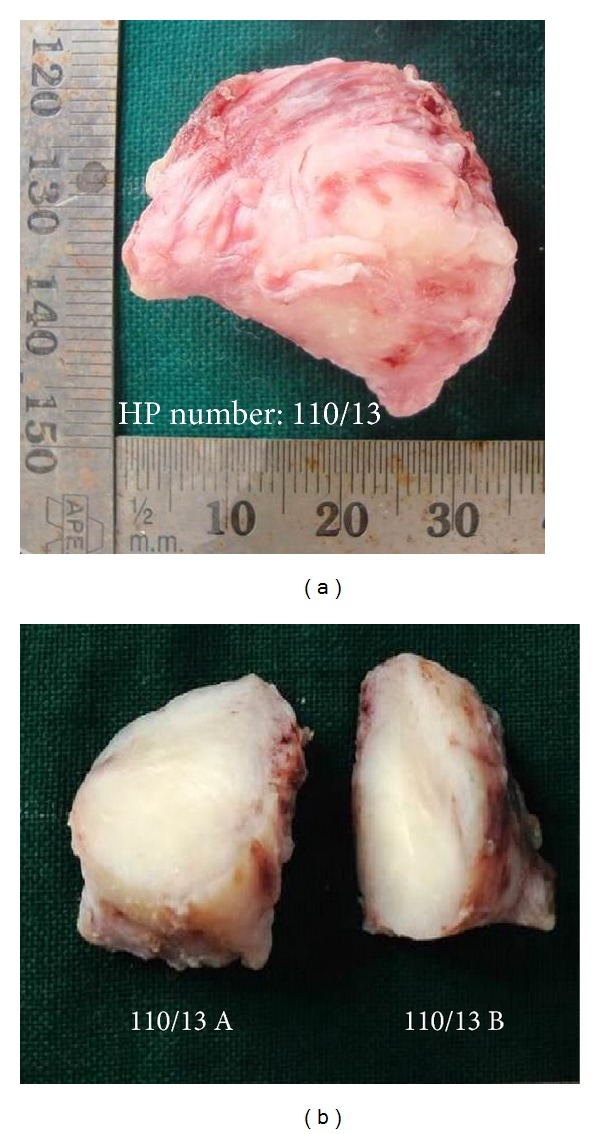
(a) Gross specimen of the excised tumour. (b) Cut surface of the excised tumour.

A soft tissue specimen, pinkish white in colour, irregular in shape, and firm in consistency, that measured approximately 3.5 × 3 × 3 cm was received (Figure 2(a)). The cut surface of the gross specimen was whitish in colour (Figure 2(b)). Difficulty in sectioning the specimen prompted that the tumour was fibrous in nature.

**Figure 3:**
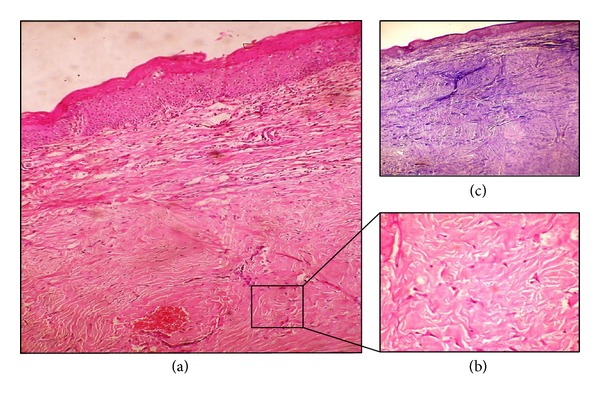
(a) H & E, 4x: well demarcated lesion with a capsule and areas of hyalinization. (b) H & E, 40x: numerous plump fibroblasts. (c) Masson trichrome, 4x: positive for collagen.

Histopathology revealed a flattened hyperparakeratotic stratified squamous epithelium overlying a well circumscribed encapsulated mass of dense collagenous stroma. The stroma was composed of numerous spindle shaped plump fibroblasts, collagen arranged in parallel or interlacing dense bundles with areas of hyalinization, sparse inflammatory cells, and minimal vascularity (Figures 3(a) and 3(b)). The tissue was further subjected to a special stain and immunohistochemical procedure. Masson trichrome stain was positive for fibrous tissue and immunohistochemistry stain S-100 was found to be negative for neural tissue (Figure 3(c)).

Thus, a diagnosis of “True Fibroma” was arrived at, eliminating lesions like irritation fibroma, peripheral giant cell granuloma, peripheral ossifying fibroma, giant cell fibroma, neurofibroma, and pyogenic granuloma.

## 3. Discussion

Localized fibrous tissue overgrowths are very common in the oral mucosa. As far as the nature of these lesions goes, most pathologists believe that both hyperplasias and neoplasms can occur. However, it is very difficult to differentiate between the two and decide whether a benign neoplasm exists or not. The extremely low frequency of occurrence of true fibromas (benign neoplasms) was highlighted by Barker et al. in 1967 when they reported two true fibromas among 171 specimens of localized fibrous growths [2, 5].

A reactive or irritational fibroma usually has an etiology, that is, a source of irritation, while benign fibrous neoplasms do not have that. Also, the character of these lesions tells a story. According to Barker and Lucas, irritational fibromas exhibit a pattern of collagen arrangement depending on the amount of irritation and the site of the lesion. There are two types of patterns: (a) radiating pattern and (b) circular pattern.

In the radiating type, the fibres radiate towards the epithelium from the base of the lesion. While the circular type shows a central mass of disoriented fibres surrounded by a peripheral layer of collagen fibres running beneath and parallel to the overlying epithelium. Thus, they hypothesized that the former appears when there is a greater degree of trauma and in sites which are immobile in nature (e.g., palate), while lesser trauma induces the latter and it occurs in sites that are flexible in nature (e.g., cheeks) [2].

True fibromas can be differentiated from their irritational counterparts on the basis of

1. the character of the collagen fibres of the lesion (does not have either of the patterns),
2. the sharp demarcation of the tissue from the surrounding normal tissue,
3. the presence of a capsule.

The present case also showed histopathological features suiting the above mentioned criteria, which pointed towards the diagnosis of a true fibroma.
